# Exploring health seeking behaviors for common cold management

**DOI:** 10.1016/j.rcsop.2023.100301

**Published:** 2023-07-11

**Authors:** Negin Keshvari, Nazila Yousefi, Farzad Peiravian, Zahra Sharif

**Affiliations:** a School of Pharmacy, Shahid Beheshti University of Medical Sciences, Valiasr St, Tehran, Iran; b Department of Pharmacoeconomics and Pharma Management, School of Pharmacy, Shahid Beheshti University of Medical Sciences, Valiasr St, Tehran, Iran; c Faculty of Pharmacy, Alborz University of Medical Sciences, Valiasr St, Shora Bvd, Alborz, Iran

**Keywords:** Common cold, Health seeking behavior, Disease management

## Abstract

**Background:**

The prevalence of common cold can impose financial burden on the healthcare systems, despite its simple and self-limiting symptoms.

**Objective:**

This study examines the behavior of patients suffering from symptoms of the common cold and explores the factors that may influence such behaviors.

**Methods:**

A descriptive-analytic cross-sectional study was conducted in 2019, in Tehran, Iran, using cluster sampling in socioeconomically diverse areas within the city. The participants' behaviors and related factors were evaluated using a 10-item questionnaire. Data collection process involved selecting 5 shopping centers and 404 individuals participated the study. SPSS version 24 was used for analysis.

**Results:**

The results showed that 42.1% of the respondents would consult a physician immediately upon experiencing cold symptoms, while 11.4% would consult a pharmacist.

In addition, 14.3% would try self-medication, 28.3% relied on traditional home remedies, and 15%indicated not to make use of any remedies or interventions. The study indicated a correlation between people's behaviors concerning the common cold and their level of health self-confidence, knowledge of the common cold, lifestyle, gender, marital status, occupational status, insurance status, and average family spending.

**Conclusion:**

The findings of this study are significant in that they shed light on the behaviors of individuals and associated factors related to seeking medical assistance for the common cold. This knowledge can assist healthcare systems in developing strategies aimed at enhancing treatment outcomes, and decreasing costs.

## Highlights

• Our understanding of people's health behavior in response to colds, is incomplete.
• This descriptive cross-sectional study examined how people behave when they have cold and what might cause them to act differently.
• Visiting a physician, resting, and self-medication were among the most favorable choices made by the respondents.
• Health confidence, knowledge about the common cold and lifestyle were among factors that can affect people's behavior.
• This study identified some gaps that can help health systems improve the effectiveness of cold management and reduce costs.

## Introduction

Despite its simple and self-limiting symptoms, the common cold can result in a financial burden on the healthcare systems, as people take time off work, visit physicians, and purchase medicines to treat symptoms. In the United States, colds are the leading cause of physician visits, with approximately 500 million episodes of acute viral upper respiratory tract infections annually, resulting in a cost of nearly $40 billion.^1^^,^^2^ According to the published evidence, almost all of the responsible pathogens causing the common cold are viruses, commonly rhinoviruses, and the most typical symptoms are rhinitis, cough, fatigue, and body aches, with a usual duration of 7–10 days.^3^ Moreover, outbreaks of colds and flu during winter, can lead to severe respiratory infections and notable deaths,^4^ placing a significant financial burden on healthcare systems.^5^ In Iran, the incidence rates of influenza-like illness were 180 and 160 per 100,000 people in 2014–2015 and 2015–2016, respectively.^6^ In developing countries like Iran, the burden of epidemics can be even more significant due to limited resources and a weak economic situation.^7^ In this situation, the irrational management of colds engaged by health professionals as well as laypersons, can produce additional problems, such as antimicrobial resistance and a decline in overall quality of life.^8^^,^^9^

Individuals who are affected by the common cold often employ a range of behaviors to alleviate their symptoms: they rest at home, increase their intake of water and soup, take nutritional supplements, use OTC (Over The Counter) medicines or prescription medications that may include antibiotics.^2^

With remarkable advancements in access to medical information and failure to comply with pharmaceutical law, which forbids selling prescription medicines without physicians' orders, people now have easy access to all the medications they seek. Therefore, self-medication with prescription drugs is becoming prevalent among various populations worldwide including Iran.^10^^,^^11^

Besides the tendency toward self-medication, other factors can influence people's health-seeking behaviors, especially in the treatment of common and less serious diseases. One such factor is the quality of the relationship between people and healthcare providers, particularly pharmacists and physicians.^12^ The impact of the patient-provider relationship on health outcomes is significant. Good relationships between patients and physicians can encourage people to be more forthcoming about their health concerns, empowering patients to make informed decisions about treatment options.^13^ As pharmacists are the most available health specialists and they advise without any charge in many countries including Iran, the role of pharmacists has changed in recent decades from dispensing medications to providing pharmaceutical care^14^; so, the patient-pharmacist relationship is becoming increasingly important in influencing patient behavior when it comes to managing minor illnesses such as the common cold.

In addition to above-mentioned factors, individuals' behaviors facing to the common cold is a reflection of their lifestyles. Despite this, there has been a lack of research on the impact of lifestyle as a variable on decision making regarding the common cold. Furthermore, previous studies highlighted various factors including demographic characterization, insurance status, knowledge about the common cold, health literacy, and health confidence that may also influence patient behavior.^1^ For example, people who have health insurance are more likely to opt for a physician's visit when face with health problems and less likely to self-treat.^15^ In addition, studies show that limited knowledge about the nature of diseases leads people to take improper medications or behave irrationally in an attempt to manage their symptoms.^2^^,^^16^ Literature has shown that low health confidence leads to more demand for healthcare services and a lower ability to self-manage health needs.^17^

As the information about the patterns of individuals' behaviors in the management of the common cold may help health policy makers to increase outcomes and reduce the cost, and because of the limited evidence regarding people's health-seeking behaviors in coping with the common cold in Iran, and researches of the variables that influence their decisions is limited, this study was designed to better understand their behavior and examine its influencing factors.

## Method

In 2019, a descriptive-analytical cross-sectional study was conducted in Tehran, the capital city of Iran. All residents of the city who visited the selected shopping centers during data collection were the study population.

## Sampling technique

A cluster sampling was employed, taking into consideration the socio-economic differences among residents of various parts of the city, so that the city was divided into 5 different zones; northern, southern, western, eastern and central regions of the city. The Cochrane formula for sample size was used to calculate the required sample size of at least 384. Cluster sampling was performed by randomly selecting one shopping center in every zone and entering participants proportionally to the size of the population living in each zone (108 samples from central and eastern zones, 80 samples from western zones, and 54 ones from northern and southern zones). Shopping centers were selected among those that marketed daily needs, including grocery stores, bakeries or department stores, to ensure that the study population was from different socioeconomic types.

The inclusion criteria included being a resident of Tehran, having the ability to make decisions regarding their health and treatment, and being between the ages of 18–65. Exclusion criteria comprised of unwillingness to participate or incomplete questionnaire responses.

## Research instrument

In order to investigate the behavior of participants when experiencing symptoms of the common cold, an overview of previous studies regarding patients' help seeking behavior and common cold management was conducted and a researcher-designed questionnaire was developed incorporating aspects from previous studies.^11^^,^^13^^,^^18^, ^19^, ^20^, ^21^

The questionnaire consisted of 10 sections and 56 questions, covering topics such as knowledge of colds and antibiotics, health confidence, the tendency to self-medicate, patient-physician relationship, patient-pharmacist relationship, and lifestyle. The first section of the questionnaire gathered demographic information, including age, sex, education level, occupation, insurance status, family size, and monthly family expenses. Subsequent sections delved into participants' health status, health confidence, knowledge of colds and antibiotics, their relationship with physicians and pharmacists, and their inclination toward self-medication. Section nine investigated lifestyle habits, while the final section examined participants' responses to cold symptoms.

To measure face and content validity, the questionnaire was reviewed by 15 experts, including 5 clinical pharmacy experts, 4 behavioral sciences experts, and 6 pharmaceutical policy experts. These experts evaluated the questionnaire based on criteria such as necessity, clarity, simplicity, and thematic relevance to the research topic. According to the number of experts, all content validity ratio (CVR) of 0.6 and above was considered valid.^22^ Based on the index introduced by Waltz & Bausell,^23^ the content validation process was carried out and items with a content validity index (CVI) of 0.79 and above were preserved. Furthermore, functionality and reliability were ensured through a pilot study. The questionnaire was subjected to 30 individuals. A satisfactory Cronbach's alpha of 0.872 was calculated (the questionnaire and scoring method are provided as the supplement file).

## Data collection and statistical analysis

A trained researcher was assigned to refer to the selected shopping centers and collect data. All participants were asked for written informed consent prior to participation in the study, with a guarantee of confidentiality for their personal information. Following data collection, the questionnaires were coded and entered into SPSS version 24 for data analysis. To ensure the security of personal data, they were encrypted and unauthorized researchers had no access to them. Tables of frequencies and percentages were used to present the results of descriptive statistics. The chi-square test was used to investigate the underlying factors that can affect the behaviors of respondents when they face common cold symptoms.

## Results

A total of 404 participants took part in this study, resulting in a response rate of 93.3%. Of the participants, 62.3% were men, 54.1% were married, and 60.3% were employed. The average monthly family expenditure was $134 or less for 29.1% of the participants, and only 1% did not provide expenditure information. It is worth noting that these expenditures correspond to the purchasing power parity (PPP), which was 29,704.31 for Iran during the year of the study.^24^ All demographic information is presented in Table 1.Health status of participants are provided in Table 2.

**Table 1:** Demographic information of participants, residents of Tehran who referred to selected shopping centers during study period, 2019.

Demographic items	Status	Number	Percent
Gender	men	251	62.3%
	women	152	37.7%
Marital status	married	216	54.1%
	single	183	45.9%
Occupation	employed	242	60.3%
	unemployed	46	11.5%
	student	53	13.2%
	retired	26	6.5%
	housekeeper	34	8.5%
Education	illiterate	14	3.5%
	high school	170	42.4%
	bachelor	150	37.4%
	master's degree	55	13.7%
	Ph.D. or more	12	3%
Insurance	no	88	22.2%
	yes	309	77.8%
Complementary insurance	no	197	52.5%
	yes	178	47.5%
Number of family members	2 or less	78	19.6%
	3 or 4	231	58.2%
	5 or 6	83	20.9%
	more than 6	5	1.3%
Average monthly cost	missing	4	1%
	$ 134 or less	116	29.1%
	$ 134–200	103	25.9%
	$ 200–300	93	23.4%
	more than $ 300	82	20.6%

**Table 2:** Health status of the participants.

Items	Status	Number	Percent
Chronic disease	no	339	84.5%
	yes	62	15.5%
Regular medicine consumption	no	313	77.5%
	yes	91	22.5%
Number of medicines (per day)	no medicine	313	79%
	1	34	8.4%
	2	21	5.2%
	3	7	1.7%
	4	8	2%
	5 and more	15	3.7

## Participant's health status

According to the results of the study, 15.5% of participants reported having a chronic disease, and 22.5% took medication regularly.

## Participants' knowledge and attitude

Self-confidence in one's health was moderate in 66.8% of the participants, while 32.3% of them had high self-confidence, and only 1% of them reported weak self-confidence. Knowledge about the common cold was moderate in 55.3% of participants and low in 14.2% of them. Additionally, in terms of knowledge about antibiotics, 76% of the participants had low knowledge (Table 3).

**Table 3:** Health self-confidence and knowledge of participants.

Variables	Good % (N)	Moderate% (N)	Weak% (N)
Health confidence	32.3% (129)	66.8% (267)	1% (4)
Knowledge about the common cold	30.5% (122)	55.3% (221)	14.2% (57)
Knowledge about antibiotics	1.8% (7)	22.3% (89)	76% (304)

The rate of self-medication was moderate in 56.3% (sometimes) and high in only 1.8% of participants (often) (Table 4). Furthermore, the health lifestyle of 76.8% of the participants was low-risk, while 2%of them had a high-risk lifestyle, and 21.2% enjoyed a healthy lifestyle.

**Table 4:** Tendency to self-medication.

Variable	Often	Sometimes	rarely
Self-medication	1.8% (7)	56.3% (222)	41.9 (165)

## Relationship quality with healthcare providers

The study found that most of the participants had moderate or weak relationships with both physicians and pharmacists (Table 5).

**Table 5:** relationship with physicians and pharmacists.

Patient relationship	Good% (N)	Acceptable% (N)	Weak% (N)
Relation with the physician	15.4% (60)	79.9% (311)	4.6% (18)
Relation with the pharmacist	5.6% (22)	88.2% (344)	6.2% (24)

## Health-seeking behavior for coping with the common cold

**Fig. 1:**
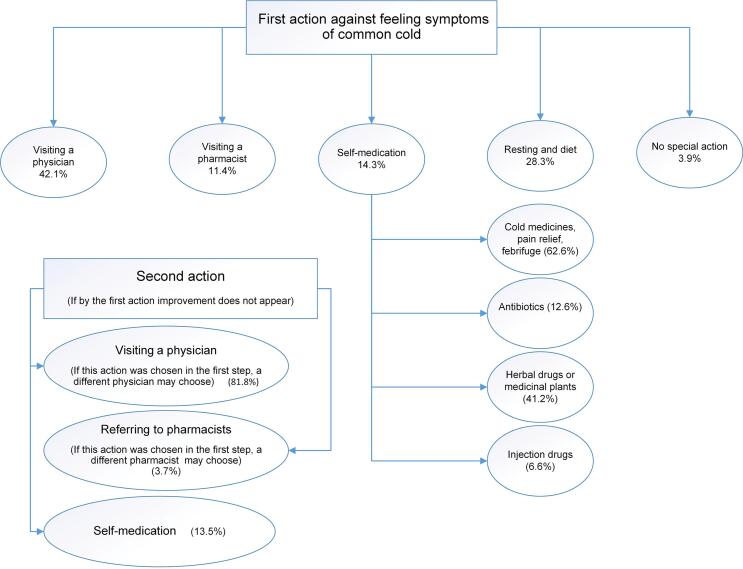
Shows the participants' first and second actions when facing symptoms related to the common cold.

The study found that when feeling the symptoms common cold, 28.3% of respondents stated that they would relax and consume hot drinks like soup or milk; 42.1% sought medical advice immediately, while only 11% would visit a pharmacist initially. Of the participants 14.3% reported treating themselves, while a small percentage of them (3.9%) believed that the common cold was a self-limiting illness and did not require a response (Fig. 1). Regarding the decision to see a pharmacist first, the survey found that 39.6% of the participants chose to do so because of lower cost and 35.6% did so because it would take less time (Table 6).

**Table 6:** Behaviors and expectations during a cold.

Questions	Options	Frequency (%)
The reason for pharmacy preference⁎	affordability of pharmaceutical care fee	39.6
	treatment received at a quicker rate	35.6
	pharmacist more easily accessible	26.8
	pharmacist usually Providing more treatment options	16.2
Choices in self-medication⁎	analgesics, fever relief, combination medicines to relieve common cold symptoms	62.6
	antibiotics	12.6
	herbal medicines (including traditional medicines)	41.2
	injectable medicines	6.6
	supplements like vitamin c	41.9
Expected time to recover	1 day	5.6
	2–3 days	40.1
	4–5 days	45.2
	More than 5 days	9.8
Second action, if no improvement occurs through the first action	visiting a doctor (if this action was chosen in the first step, another doctor can be chosen).	81.8
	referring to a pharmacist (if this action was chosen in the first step, another pharmacist could be chosen).	3.7
	self-medication	13.5

⁎ Respondents could choose more than one item.

In the case of self-medication, most of the respondents (63.9%) reported that they would choose OTC medications such as paracetamol. Herbal medicines were the first choice for a rather large number of the participants (41.2%), and 12.6% of the participants indicated that they would use antibiotics to self-medicate.

Regarding the duration of the common cold, 85.3% of the respondents believed that they would heal within 2 to 5 days; while 9.2% thought it would take longer for them, and a mere 5.6% expected that their colds would go away in just one day.

If the participants did not feel better after taking their first action, 82.8% of participants would visit a doctor, and 13.5% would opt for self-medication. Only 3.5% said they would visit a pharmacist for help with this problem.

## Underlying factors

In order to identify underlying factors that can affect the behaviors of respondents when they feel common cold symptoms, chi-square test was done. The result of the cross-tabulation and chi-square test revealed a significant correlation (with a significance level of less than 0.05) between the first action taken for common cold and demographic characteristics such as marital and occupation status, insurance coverage, health confidence, the tendency to self-medication, knowledge about the common cold, lifestyle (0.008), and relationship with physicians. In other words, changes in these variables lead to change the help seeking behavior of participants. The other factors such as gender, level of education and knowledge about antibiotics had no significant correlation with respondents' behaviors (Table 7).

**Table 7:** Correlation between first actions taken for common cold.

Variable	Significance level (*P* value)
Gender	0.125
Marital status	0.001
Occupation	0.000
Education	0.929
Average monthly cost	0.500
Insurance coverage	0.006
Health confidence	0.000
Self-medication	0.000
Knowledge of common cold	0.000
Knowledge of antibiotics	0.114
Patient-physician relationship	0.040
Patient-pharmacist relationship	0.417
Health lifestyle	0.002

## Discussion

This study examined how individuals behave when they encounter the common cold and recognized the factors that may impact their action. Studies show that colds and fevers are the most common health complaints that increase absenteeism and reduced staff capacity, leading to large financial burdens on companies.^25^ Despite its importance, our understanding of people's responses to it is incomplete.

According to the study results, a large number of participants (about 42%) immediately try to see a physician if they had a cold, Cost of visiting a physician is not limited to visit cost, and physicians usually prescribe a list of medicines to satisfy their patients.^26^ However, the common cold is a viral infection, and there is no specific treatment should recommend by physicians for viral colds.^27^ Higher levels of knowledge about common colds among the participants or referring to pharmacists could reduce physicians' workload and costs in this area. Although knowledge about the common cold significantly influences participants' decision making against the common cold, only 30% of participants had good knowledge, and only 11% of them ask from pharmacists. These results are consistent with the findings of Bliass et al. from the U.S. in 2015, who indicated that there is a gap between respondents' knowledge about common colds and appropriate knowledge. They also found that almost a quarter of people believe in antibiotics as an appropriate treatment for common colds^28^ which the current study similarly shows weak knowledge of antibiotics in most participants. This fact previously was shown by Hosseinzade et al. in Iran, that the general public's knowledge about antibiotics is inadequate and most individuals take them without a prescription.^29^

Evidence shows that symptoms of a cold last for 7–10 days on average^2^ and may last for three weeks on some occasions.^9^ However, in our study, almost half of the respondents expected their symptoms to disappear within 3 days. This expectation which is due to low knowledge might lead most of them to visit a physician as their first and second course of action. Similarly, with a small difference, respondents in the U.S. expected relief of symptoms after 1 to 6 days.^28^

Prior studies have shown an association between low health confidence and demand for health services.^30^ Chi-square testing showed the significant effect of health confidence on help-seeking behavior of respondents. In this study, about 28% of the participants preferred rest and home care to treat their cold, which may be related to their high and moderate health confidence. In other words, knowledge about the common cold is not enough for shaping health behaviors and self-confidence in decision making. Accordingly, policy makers should plan to improve public health confidence as well as their health knowledge. Moreover, discrepancies between knowledge and practice in the treatment of the common cold may be attributable to inappropriate translation of knowledge into behavior. Future studies of the public's translation of knowledge about mild health problems such as common colds into rational behavior could reduce the burden of these common but not serious problems on healthcare systems.

Although people need to feel confident to manage their health^17^, health overconfidence may lead to self-medication. Studies show a high prevalence of self-medication in Europe, African countries, and Iran. The prevalence of self-medication in Iran is three times greater than the global average, with 83.3% of Iranians reportedly willing to self-medicate.^31^ In addition, cold, cough, pharyngitis, and bronchitis were reported as the most prevalent ailment for which people self-medicate in previous studies.^27^^,^^32^^,^^33^ In our study, although participants generally reported a low to moderate tendency to self-medicate (98.2% of respondents), they usually used cold tablets (a general name for multi-component tablets usually include paracetamol, chlorpheniramine, and phenylephrine), painkillers, and feverfew pills to self-medicate for the common cold. This outcome is also confirmed in a study conducted in 2018 by Abdi et al.^28^ among medical students in Kermanshah, showing that the most commonly used drugs for self-medication were cold tablets, paracetamol, and antibiotics such as amoxicillin. Based on the findings of this study, the second most prevalent form of self-medication was herbal medicines such as herbal cough syrups or medicinal plants. The high consumption of herbal medicines might result from the misconception that taking herbal medicines have acceptable efficacy with few side effects.^34^ Similarly, people might have a misconception about the effectiveness of antibiotics for common colds,^35^ leading to the indiscriminate use of these medicines. While it is illegal to dispense antibiotics without a prescription in Iran, many pharmacies illegally continue to sell antibiotics without a prescription and even without a pharmacist consultation. This would increase the rate of self-medication with antibiotics in Iran.

According to the literature, although pharmaceutical care is popular in many regions around the world, in some countries, including Iran, it has not been becoming a mature concept.^36^ The accessibility of pharmacists in the community makes them a potential first course of action for minor illnesses like the common cold, replacing referring physicians. The public can benefit from the expertise of pharmacists in medication issues and trust them to provide appropriate treatment. Although participants declare that going to the pharmacy involved less time and expenses for them, in practice they preferred physician as the first and second action for treating a common cold. In this study, the preference for self-medication is even higher than seeking pharmacists' consultation, possibly due to a lack of awareness of pharmacists' competencies and responsibilities. Although, people may view pharmacies as salesman delivering medications,^37^ using pharmacists as the initial point of care can lead to cost savings and better health outcomes for minor illnesses like the common cold.

This study was the first attempt to examine people's health seeking behavior regarding the common cold in Iran and try to shed light on some critical health issues which are increasing health system costs. As not attention to the common cold symptoms and self-medication may lead to the prolongation of disease, productivity loss, increasing cost of other complications, decreasing quality of life, over attention and visiting physicians as the first attempt action may cause some unnecessary costs, such as cost of transportation, visit, time expending in physician offices, prescription, and even unnecessary medications.

Increasing people's knowledge about self-limiting diseases such as the common cold, increasing their health confidence, managing their overconfidence which may lead to self-medication, introducing pharmacists to the community as a valuable actor in the health system may lead to higher health achievement with lower cost.

The burden of the common cold on the healthcare systems could have been reduced if pharmacists are well equipped with the knowledge to manage mild ailments, and regulation support their interventions. Awareness of the pharmacist's role in treating the common cold should be considered in health campaigns to reduce the burden on healthcare systems.

Finally, interference of findings of this study should be done with some consideration regarding its limitation. Same as all self-reported surveys, the response may reflect respondents' values instead of their actual tendencies or behaviors. Other limitations include recall bias as respondents may not face the common cold in recent months. The population participating in this study as well as study time frame are limited and it may limit the result generalization to other times and places. Moreover, as the help-seeking behavior of patients and underlying factors were not studied before, this study tried to simply identify effective variables. The strength of the association between individuals' behavior and variables, and the prediction of behavior based on identified variables, were not analyzed in this study.

## Conclusion

The management of common colds and the health-seeking behaviors of the importance of health seeking behavior of individuals in the management of the common colds have been neglected by healthcare decision-makers, despite the substantial burden it places on healthcare systems. If it is not well regarded, it may lead to poor health achievements and a waste of money. This study sought to identify people's health seeking behaviors in managing their common cold and an the results revealed that the most prevalent and preferred options for managing colds were visiting a physician, and resting without asking for any advice from healthcare providers. According to our knowledge, the common cold is a self-limiting disease which does not need to immediately visit a physician. The tendency to visit physicians in these cases may increase health system workload and costs. On the other hand, doing nothing or self-medication may lead to health complications and further costs. Our suggestion for achieving higher outcomes with lower costs is more attention to pharmacists' abilities for managing simple diseases. Health policy makers can improve pharmacists' role in managing mild diseases by empowering pharmacists and educating the general public about pharmacists' roles and abilities. Furthermore, according to the findings of this study, increasing people's knowledge regarding the common cold, increasing their health confidence, and avoiding self-medication, may lead to higher health achievements with lower costs in the common cold management.

## Funding

This research did not receive any specific grant from funding agencies in the public, commercial, or not-for-profit sectors.

## Declaration of Competing Interest

The authors declare that they have no known competing financial interests or personal relationships that could have appeared to influence the work reported in this paper.

## Appendix A Supplementary data

Supplementary material Image 1
