# Supplementary material for: Exploring health seeking behaviors for common cold management
Source: Explor Res Clin Soc Pharm. 2023 Jul 11;11:100301. doi: 10.1016/j.rcsop.2023.100301 (PMC10392600; doi:10.1016/j.rcsop.2023.100301)
Supplement: Supplementary file 1 — Supplementary material [file mmc1.docx]

| **Demographic information** | |
| --- | --- |
| Gender: male female | Marital status: Married Single |
| Age: | Occupation: Employed Unemployed Homemaker Student Retired |
| Education: Illiterate High School Diploma or Below Bachelor’s Master’s Ph.D. | |
| Insurance:  Basic Insurance: Yes No  Complementary Insurance: Yes No | |
| Number of Family Members: Two or less Three or four Five or six Six or more | |
| Family's Average Monthly Expenditure: $500 or less $500 - $800 $800 - $1100 More than $1100 | |

| **Health Condition:** |
| --- |
| Do you have any chronic diseases? (e.g., diabetes, respiratory illness, heart disease) Yes No |
| Do you take any medications regularly? Yes No |

| Strongly disagree | Disagree | Neutral | Agree | Completely agree | **Health Confidence**  Please rate your level of agreement with the following statements regarding your health. Please choose the option that best reflects your opinion for each statement: | |
| --- | --- | --- | --- | --- | --- | --- |
|  |  |  |  |  | I am knowledgeable about my current health status. | 1 |
|  |  |  |  |  | I know how to take care of myself when I am suffering from common illnesses. | 2 |
|  |  |  |  |  | I am able to access the necessary medical treatment when I need it. | 3 |
|  |  |  |  |  | My healthcare providers seek and value my opinion about my medical care. | 4 |

| Yes | I don’t know | No | **Knowledge about the common cold** | |
| --- | --- | --- | --- | --- |
|  |  |  | Can the common cold heal on its own? | 1 |
|  |  |  | Is the common cold a contagious disease? | 2 |
|  |  |  | Do you believe that the common cold and influenza are the same illness? | 3 |
|  |  |  | Can consuming soup, warm liquids, and citrus fruits help to cure a common cold? | 4 |
|  |  |  | Do you think it is necessary for people to visit a doctor when they catch a cold? | 5 |
|  |  |  | Do you think people should know how to treat themselves when they catch a cold? | 6 |
|  |  |  | Can the common cold cause death? | 7 |
|  |  |  | Can antibiotics cure a common cold? | 8 |
|  |  |  | Which of the following options can transmit the common cold? | 9 |
|  |  |  | Sneezing |  |
|  |  |  | Coughing |  |
|  |  |  | Direct contact with an infected person |  |
|  |  |  | Dust |  |
|  |  |  | Sharing personal things |  |
|  |  |  | Poor hygiene |  |
|  |  |  | Which of the following can be side effects of the common cold? | 10 |
|  |  |  | Sinusitis |  |
|  |  |  | Ear inflammation |  |
|  |  |  | Headache |  |
|  |  |  | Vomiting |  |
|  |  |  | Sore throat |  |
|  |  |  | Diarrhea |  |
|  |  |  | Fever |  |

| incorrect | I don’t know | correct | **Knowledge about antibiotics** |
| --- | --- | --- | --- |
|  |  |  | Viruses can be cured by antibiotics. |
|  |  |  | Antibiotics are effective in treating the common cold and influenza. |
|  |  |  | Unnecessary usage of antibiotics can lead to ineffectiveness. |
|  |  |  | Antibiotic use can result in side effects such as diarrhea. |
|  |  |  | Antibiotics should not be used as anti-inflammatory drugs. |
|  |  |  | It is illegal to sell antibiotics without a prescription. |

| Completely Correct | Often Correct | Correct | Somehow  Incorrect | Completely Incorrect | **Relationship with the physician**  Please rate the following statements based on your relationship with your physician: | |
| --- | --- | --- | --- | --- | --- | --- |
|  |  |  |  |  | Physicians are helpful to me. | 1 |
|  |  |  |  |  | Physicians have enough time for me. | 2 |
|  |  |  |  |  | I trust my physicians. | 3 |
|  |  |  |  |  | Physicians understand my medical problems. | 4 |
|  |  |  |  |  | I am able to explain my health problems to my physician. | 5 |
|  |  |  |  |  | I am satisfied with the treatment provided by my physician. | 6 |
|  |  |  |  |  | Physicians are easily accessible to me. | 7 |
|  |  |  |  |  | I am confident that my common cold will be cured if I visit my physician. | 8 |
|  |  |  |  |  | The cost of visiting my physician is a factor in my decision-making process. | 9 |

| Completely Correct | Often Correct | Correct | Somehow Incorrect | Completely Incorrect | **Relationship with the pharmacist**  Please rate the following statements based on your relationship with your pharmacist: | |
| --- | --- | --- | --- | --- | --- | --- |
|  |  |  |  |  | Pharmacists are helpful to me. | 1 |
|  |  |  |  |  | Pharmacists have enough time for me. | 2 |
|  |  |  |  |  | I trust my Pharmacists. | 3 |
|  |  |  |  |  | I am able to explain my health problems to my physician. | 4 |
|  |  |  |  |  | Pharmacists are easily accessible to me. | 5 |
|  |  |  |  |  | Pharmacists understand my explanations. | 6 |
|  |  |  |  |  | The knowledge of pharmacists is enough to improve my level of health. | 7 |
|  |  |  |  |  | Pharmacists should give me every drug that I want. | 8 |
|  |  |  |  |  | Pharmacists have the ability to advise me on my medical problems and make decisions regarding my medication. | 9 |
|  |  |  |  |  | I appreciate receiving necessary advice from pharmacists regarding my medical problems. | 10 |
|  |  |  |  |  | Based on my previous experiences, I am encouraged to consult with pharmacists about my medical problems. | 11 |
|  |  |  |  |  | I am confident that my common cold will be cured if I visit a pharmacist. | 12 |

| Completely Correct | Often Correct | Correct | Somehow Incorrect | Completely Incorrect | **Self-medication** | |
| --- | --- | --- | --- | --- | --- | --- |
|  |  |  |  |  | I am able to use the same prescription for the same symptoms without consulting the doctor again. | 1 |
|  |  |  |  |  | I am knowledgeable about my body and know which drugs to use for minor illnesses. | 2 |
|  |  |  |  |  | I believe that my friend's experiences with visiting the doctor or having a similar disease can be a useful reference for choosing treatment and medication for my own disease. | 3 |

| Never | Rarely | Sometimes | Often | Almost Always | **Lifestyle**  Please rate the following statements based on your lifestyle habits: |
| --- | --- | --- | --- | --- | --- |
|  |  |  |  |  | I read the information on food labels. |
|  |  |  |  |  | I follow a healthy diet. |
|  |  |  |  |  | I engage in regular exercise. |
|  |  |  |  |  | I exceed the speed limit when driving. |
|  |  |  |  |  | I avoid using tobacco products like cigarettes. |

| **Behavior during a common cold**  Please select one of the following statements based on your behavior during a common cold: | |
| --- | --- |
| How do you typically manage a cold?  a) I visit a physician immediately.  b) I visit a pharmacist (pharmacy) immediately.  c) I self-medicate without consulting a doctor or pharmacist.  d) I don't seek medical help and manage my symptoms by resting and consuming food like soup, warm liquids, and citrus juice.  e) I do not take any action and wait for the cold to resolve on its own due to a busy schedule. | 1 |
| Why do you prefer visiting a pharmacist over a physician? (Select all that apply.)  a) It is less expensive.  b) It takes less time.  c) Pharmacies are more accessible than doctor's offices.  d) More options are available to choose from. | 2 |
| Which medications do you typically use for self-medication? (Select all that apply.)  a) Cold tablets, painkillers, and fever pills.  b) Antibiotics.  c) Herbal medicines such as cough syrup or herbal tea.  d) Injectable drugs like dexamethasone or penicillin.  e) Vitamin C. | 3 |
| How long do you expect it to take to recover from a cold?  a) One day.  b) Two or three days.  c) Four or five days.  d) More than five days. | 4 |
| If your symptoms persist after your initial course of action, what would you do for your next course of action?  a) Visit a physician (if I previously visited a physician, I will see a different physician).  b) Visit a pharmacist (if I previously visited a pharmacist, I will visit a different pharmacy).  c) Try a different medication based on advice from friends or past experiences. | 5 |

To score the questionnaire, we use the following methods:

**Self-confidence Part:**

Strongly Agree: 5 points

Agree: 4 points

Neutral: 3 points

Disagree: 2 points

Strongly Disagree: 1 point

**Knowledge about Common Cold Part:**

No: 0 points

Neutral: 1 point

Yes: 2 points

**Knowledge about Antibiotics Part:**

No: 0 points

Neutral: 1 point

Yes: 2 points

**Relationship with the Physician, Relationship with the Pharmacist, and Self-Medication Parts:**

Completely Correct: 5 points

Often Correct: 4 points

Correct: 3 points

Somehow Incorrect: 2 points

Completely Incorrect: 1 point

**Lifestyle Part:**

Almost Always: 5 points

Often: 4 points

Sometimes: 3 points

Rarely: 2 points

Never: 1 point

After scoring each part, we divide the scores into three categories (low, medium, and high) based on expert opinion.

| lable | score | parts |
| --- | --- | --- |
| Weak | 0-8 | Health confidence |
| Moderate | 8-16 |  |
| Good | 16-20 |  |
| Weak | 0-20 | Knowledge about  the common cold |
| Moderate | 20-30 |  |
| Good | 30-40 |  |
| Weak | 0-6 | Knowledge about antibiotics |
| Moderate | 6-10 |  |
| Good | 10-12 |  |
| High risk | 0-8 | Life style |
| Low risk | 8-16 |  |
| Healthy | 16-20 |  |
| rarely | 1-2 | self-medication (based on mean score) |
| Sometimes | 2-4 |  |
| Often | 4-5 |  |
| weak | 1-2 | Relationship with the pharmacist and physician (based on mean score) |
| Acceptable | 2-4 |  |
| Good | 4-5 |  |
